# Differential gene expression during early development in brains of wildtype and biotinidase-deficient mice

**DOI:** 10.1016/j.ymgmr.2016.09.007

**Published:** 2016-10-08

**Authors:** Christian Brigolin, Nathan McKenty, Kirit Pindolia, Barry Wolf

**Affiliations:** a Department of Research Administration, Henry Ford Health System, Detroit, MI 48202, United States; b Center for Molecular Medicine and Genetics, Wayne State University School of Medicine, Detroit, MI 48201, United States

**Keywords:** Biotinidase deficiency, Gene transcription, Gene expression, Neurological abnormalities, Mouse, Knock-out mouse, Transgenic mouse

## Abstract

Biotinidase deficiency is an autosomal recessively inherited disorder characterized by neurological and cutaneous abnormalities. Untreated individuals with biotinidase deficiency cannot recycle biotin from biocytin (N-biotinyl-ϵ-lysine), the proteolytic digestion product of protein-bound biotin. Biotin therapy can markedly resolve symptoms, or can prevent the development of symptoms if initiated early. To understand better the pathogenesis of the neurological problems in the disorder in humans, we have compared gene transcription changes during the first week post-birth in the brains of biotinidase-deficient, transgenic, knock-out mice at days 1 and 8 and compared to changes in wildtype mice at the same times. The knockout pups that were not supplemented with unconjugated biotin became symptomatic by day 8 and exhibiting failure to thrive. Wildtype pups remained asymptomatic under the same experimental conditions. We compared all four possible combinations and noted the most significant up- and down-regulated genes in the knockout animals at Day 8 compared to those at Day 1, reflecting the changes in gene expression over the first week of development. These alterations involved neurological development and immunological function pathways and provide some clues to avenues for further research. At this time, these preliminary analyses provide us with limited, but new information. However, with the development of new algorithms and programs examining various mechanisms and pathways in the central nervous system, these analyses may help us to understand better the role of biotinidase and the pathogenesis of biotinidase deficiency.

## 1 Introduction

Human carboxylases are important for amino acid catabolism, fatty acid synthesis, and gluconeogenesis [1]. Biotinidase (EC 3.5.1.12) is responsible for cleaving and recycling biotin from its bound forms, such as biocytin and biotinyl-peptides [2]. Untreated individuals with profound biotinidase deficiency (< 10% of mean normal serum activity) can exhibit neurological symptoms, including hypotonia, seizures, ataxia, optic atrophy, sensorineural hearing loss, spastic paraparesis, and developmental delay [3]. Individuals with profound biotinidase deficiency (OMIM #253260) improve markedly with oral supplementation of free, unconjugated biotin, but usually continue to exhibit irreversible neurological abnormalities [4]. Early treatment with biotin can prevent the development of symptoms in enzyme-deficient individuals. This is why biotinidase deficiency has been added to the newborn screening programs of all states in the United States and in many countries [5].

We developed and characterized a transgenic, knock-out mouse with biotinidase deficiency to various study aspects of the disorder, including the neurological features [6], [7]. The clinical and neurological abnormalities observed in symptomatic mice with biotinidase deficiency are similar to those seen in symptomatic children with the disorder [3]. Biotinidase-deficient mice (KO) made symptomatic by restriction of biotin intake performed poorly on neurological tests compared to similarly treated wildtype (WT) mice. The symptomatic animals exhibited ventriculomegaly, demyelination, axonal degeneration and corpus callosum compression [7].

## 2 Methods

### 2.1 Study design

Untreated KO pups develop symptoms by 8 days post-birth, and have a typical lifespan of 8–12 days [6], [8]. Injection of biotin into the nursing dam prevents the development of symptoms in these knock-out pups. Both WT and KO dams were bred on a normal, biotin-containing diet (Fig. 2). WT pups born and nurtured by WT dams exhibit normal development throughout the study period. KO pups are born and nurtured by KO dams that do not receive biotin injections. By day eight, these KO pups fail to thrive and become symptomatic, with death occurring between days eight and twelve. The rapid development of these symptoms indicates that the KO dams fail to provide free, unconjugated biotin to newborn pups through lactation. The animals and their brains in each study group did not noticeably change in size or weight during the short eight-day study period. We have previously reported morphological parameters, biochemical characteristics and neurological features of asymptomatic and symptomatic KO mice [6], [7].

Total transcriptomes were prepared from the brains harvested from mice with both genotypes, WT and KO, at one day and at eight days of age. After determining that we had satisfactory quality control of the RNA samples, we analyzed the differential gene expression data.

### 2.2 Harvesting brains and isolating RNA

WT and KO dams were maintained on a normal, biotin-containing diet. KO pups born to KO dams were nurtured by their mother and did not receive any additional unconjugated biotin. Similarly, WT pups born to WT dams were nurtured by their mother and did not receive any additional unconjugated biotin. The KO pups failed to thrive, became symptomatic, and died in 8 to 12 days under these conditions. On the other hand, WT pups fed by their mother thrived and remained asymptomatic over this same time period.

The pups were sacrificed and their brains were harvested and stored in RNAlater (Termo Fisher, Waltham, MA) at − 80 °C for subsequent RNA isolation, as described previously in the manual provided with the kit. RNA was isolated using the miRNeasy mini kit (Qiagen) from each of the 12 stored brains, and samples were quantitated by Nanodrop (Wilmington, DE). Quality was assessed by running samples on Agilent TapeStation and using RNA Integrity Number (RIN-Agilent 2200 TapeStation). 500 ng of RNA from the three pups in each of the four groups (WT Day 1, KO day 1, WT Day 8, and KO Day 8) were pooled and subjected to Gene Expression Analysis on Agilent platform.

### 2.3 Gene expression analysis

Differential gene expression of RNA samples was compared in each of the four arrays using Agilent Mouse Gene Expression V.2 4X44K array (Santa Clara, CA). Each chip is capable of detecting for 39,430 Entrez Gene mRNAs. The quality of the results were assessed using positive control probes and spike-in control probes 31 × 10 E1A (Agilent Technologies, Santa Clara, CA) at the Functional Genomics and Bioinformatics Core Facility at Wayne State University, Detroit, MI. These probes establish baseline criteria against which experimental signals were compared. Agilent's microarray design is based on two-color microarray-based gene expression analysis (quick amp labeling) with Tecan HS Pro Hybridization. To obtain fluorescent intensities for each spot on the array, the Agilent dual laser scanner with SureScan High Resolution Technology was used.

### 2.4 Biotinidase transcript

In preparing a transgenic, knock-out mouse with biotinidase deficiency, part of intron 1, all of exon 2, intron 2, exon3 and part of intron 3 of the biotinidase gene, *BTD*, were deleted. The promoter of *BTD* was intact, and transcription of the gene thus resulted in a truncated, but detectable transcript. However, the transcript resulted in no biotinidase protein on western blot analysis against *anti*-biotinidase antibody and, therefore, no biotinidase activity in the preparation [6].

In the expression studies, a *BTD* probe was used that hybridizes to within the 3′-region of the transcript for the remainder of intron 3 and exon 4. Based on the results found in KO Day 1 and WT Day 1 mice, it appears that there is about 50% of the transcript in KO mice compared to that of WT mice. This may indicate that the transcript in the KO mice is less stable.

### 2.5 Statistical analysis

**Fig. 1:**
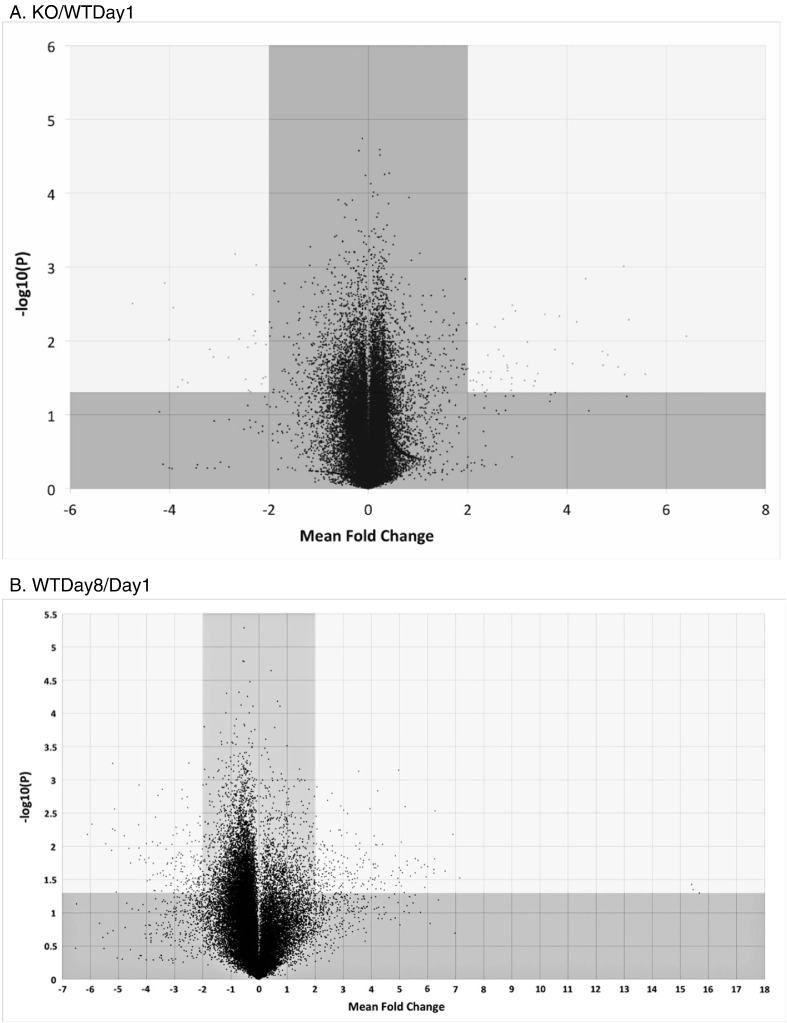

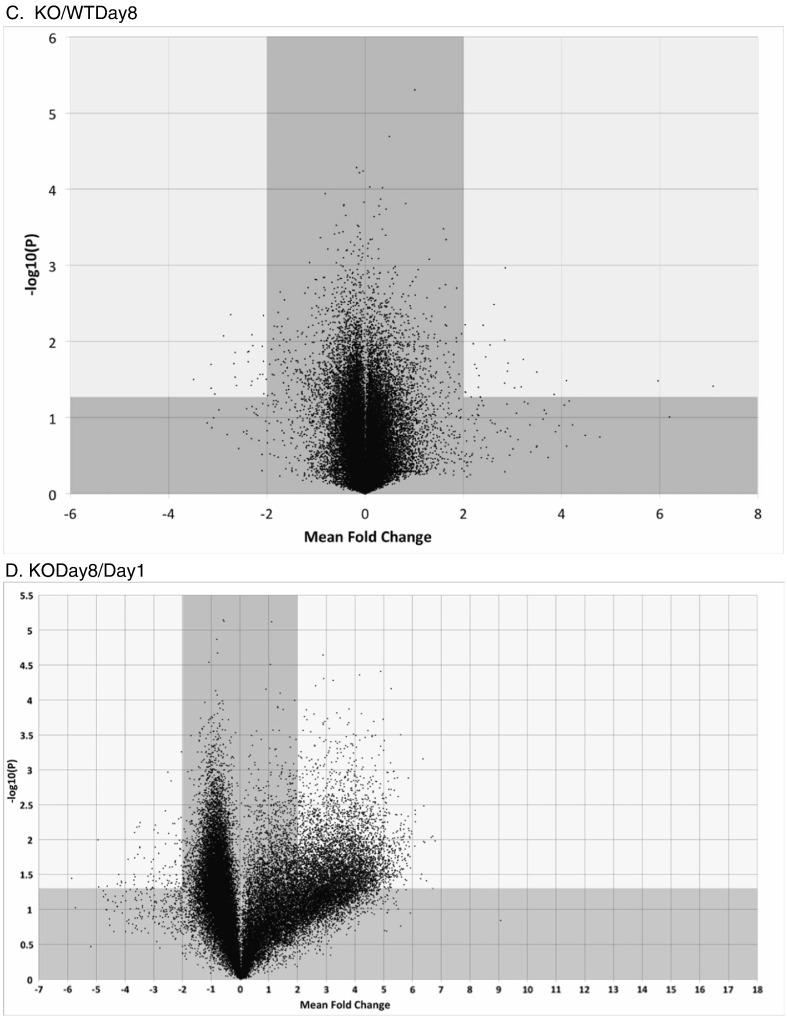
Overview of the four transcriptome signal comparisons: A. KO/WT Day1, B. WT Day 8/Day 1, C. KO/WT Day 8 and D. KO Day 8/Day 1. Volcano-plots display mean fold change (x-axis) vs. Statistical Significance (*− log10(p-value)*) (y-axis) for mRNA transcript signals. Experimentally significant probe signals shown within green regions were defined as having a mean fold change of >|2 |, and a p-value of <* 0.05* (where −* log10(P)* = *1.301*). Shaded regions indicate mRNA signals not meeting significance criteria. Note that the axes of Figs. B and D are different from Figs. A and C to accommodate the few out-lying values.

Dye-swapped duplicate data were mean averaged to control for color biases, and this value was used for subsequent analyses (GeneSpring v12, Agilent Technologies, Santa Clara, CA). The absolute fold change was converted by a (log2) transformation. *p*-value was then calculated for these fold changes, reflecting the probability of obtaining a given fold change for a probe by chance, and incorporated the control measurements from the spike-in controls and dye-swap variation. In Fig. 1, *p*-values were normalized by a (− log10) conversion. mRNA signals required a mean fold change of >|2 |, and a p-value of < 0.05 in order to be considered experimentally significant.

### 2.6 Whole transcriptome analysis

Processed data sets were analyzed using DAVID (https://david.ncifcrf.gov/(David Bioinformatics Database, National Institutes of Allergy and Infectious Diseases, National Institutes of Health, Bethesda, MD) and using Ingenuity Pathway Analysis (http://www.ingenuity.com/products/ipa) to identify functional annotations, pathways, and themes.

## 3 Results

Our study design allowed us to evaluate and compare four different differential expression scenarios. The following are the comparisons and their respective rationales:

1. Comparisons of expression of KO pups at day 1 with WT pups at day 1 (KO/WT Day 1) examine the baseline differences in gene transcription between KO and WT animals.
2. Comparisons in expression of WT pups at day 8 with WT pups at day 1 (WT Day 8/Day1) examine the differences in gene transcription of WT animals during the early developmental phase post-birth. KO animals develop symptoms during this time period, whereas WT animals remain asymptomatic. This scenario compares developmental gene changes from Day 1 to Day 8, but not changes due to the decreased intake of biotin, because these animals remain asymptomatic and have biotinidase capable of recycling endogenous biotin.
3. Comparisons of expression of KO pups at day 8 with WT pups at day 8 (KO/WT Day 8) examine differences in gene transcription due to biotinidase deficiency. In this period, KO animals become symptomatic, whereas WT animals remain asymptomatic.
4. Comparisons in expression of KO pups at day 8 with KO pups at day 1 (KO/WT Day 8) examine the differences in gene transcription of KO animals when they become symptomatic versus asymptomatic KO animals at baseline representing the developmental phase affected by biotinidase deficiency. Not only does this scenario compare developmental gene changes from Day 1 to Day 8, but also genes that are up- and down-regulated in response to the stresses of biotinidase deficiency and consequential biotin deficiency.

First, we performed an overview of the entire complement of transcriptomes for each of the above comparisons (Fig. 1). These volcano-plots or scatter-plots show the degree and probabilities of up- and down-regulating genes in each of the above comparisons. The plots display mean fold changes in expression (x-axis) vs. statistical significance (− log10(*p*-value)) (y-axis) for mRNA transcript signals. Note that the differences in magnitude of both the x- and y-axes are to accommodate major out-lying values. Significant probe signals shown within the non-shaded regions were defined as having a mean fold change of >|2 |, and a p-value of < 0.05 (where − log10(P) = 1.301). Shaded regions indicate mRNA signals that do not meet significance and magnitude criteria.

In general, most of the genes expressed in KO/WT Day 1 showed few differences in up-regulations or down-regulations which represents baselines for both the KO and WT animals, whereas there are more differences noted in WT Day 8/Day 1 which may be indicating changes in development or other processes. Because the WT animals are able to recycle biotin, it unlikely that the differences represent differences in expression due to biotin restriction or related metabolic pathways. Interestingly, there are fewer changes noted in KO/WT Day8. This is possibly because many of the gene differences are likely common to developmental processes. However, there are many more differences in gene expression noted in the KO Day8/Day1 that are comparing the differences when the KO animals are symptomatic. The changes may be indicative of major, mostly up-regulatory transcription changes in response to the metabolic stresses caused by their secondary, biotin-deficient status, and that deficiency's effects on other metabolic pathways.

**Fig. 2:**
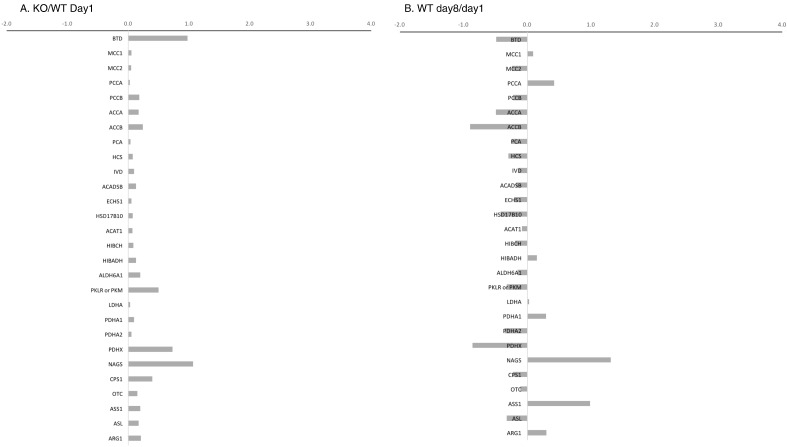

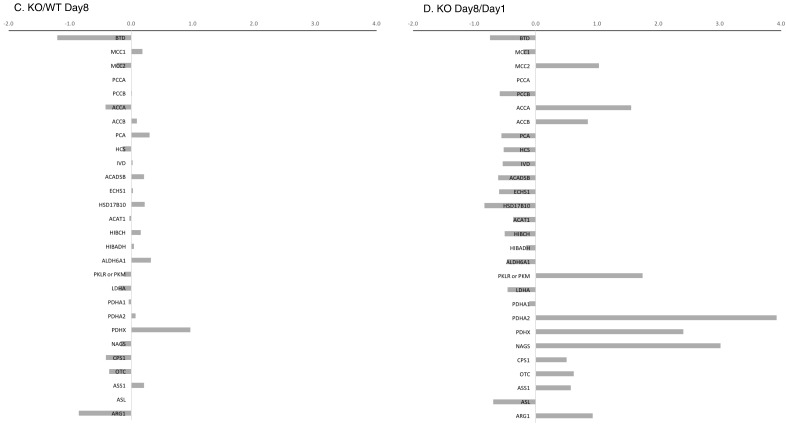
Mean fold change of selected biotin-dependent enzymes, enzymes involved with carboxylase metabolites and urea cycle enzymes for all four comparison scenarios: A. KO/WT Day1, B. WT Day 8/Day 1, C. KO/WT Day 8 and D. KO Day8/Day 1.

However, these plots only provide a comparative overview of metabolic activity differences under the various conditions and do not address specific gene changes. To evaluate specific genes, we have specifically chosen to examine the up-regulations and down-regulations of genes of the biotin-dependent carboxylases and in pathways related to their specific metabolites and those involved with the urea cycle, because individuals with untreated biotinidase deficiency often exhibit hyperammonemia (Fig. 2). As expected, *BTD*, a constitutive enzyme, did not up- or down-regulate significantly in any of the four scenarios. The various enzymes did not significantly vary more than a fold up- or down-regulation of expression in any of the four comparisons, except in the Day8/Day1 comparison for up-regulation of between 2- and 3-fold of pyruvate dehydrogenase complex (PDA2 and PDHX) and the in the enzyme responsible for the initial enhancer step in the urea cycle, *N*-acetylglutamate synthetase.

In Table 1, we examined the integrative pathways with significant up- and down-regulation expression under each of the four scenarios using Ingenuity Pathway Analysis (IPA) software. In the KO/WT Day1 comparison, there are only a few gene groupings that are up-regulated in the KO compared to WT. These genes are principally involved with brain development. Similarly, in the WT Day8/Day1 comparisons, most of the significant up- and down-regulated genes involve growth or formation of neurological structures. In the KO/WT Day 8 comparisons, there are multiple up- and down regulations of genes involved in the development of neurological structures with some genes implicated in abnormal morphology development. In the KO Day 8/Day 1 comparisons, there are multiple significant changes in functional genes, such as in excitation of brain cells/neurons and action potentials, but also alterations in immunological functions, such leukocyte movement, lymphocyte migration, and the movement of T-cell lymphocytes.

## 4 Discussion

Symptomatic mice with biotinidase deficiency developed neurocutaneous abnormalities similar to the clinical features seen in children with untreated profound biotinidase deficiency [3]. With the advent of newborn screening of biotinidase deficiency, our opportunity to evaluate these neurological is limited [5]. In order to understand better the effect of biotinidase deficiency in the mechanism and pathophysiology of the neurological changes in the disorder, we performed a preliminary study to evaluate differential gene expression in the biotinidase-deficient, transgenic mice under various comparison scenarios.

Of the four gene transcription comparisons examined, the most important and revealing appears to be the comparison of KO mice at day 8 compared to those at day 1. In this scenario, the KO animals at day 8 are markedly symptomatic, whereas the KO mice at day 1 are asymptomatic. Under these conditions, the major differences in gene expression are most likely to be the direct effect of the enzyme deficiency and the resultant biotin deficiency [6]. Most of the significantly affected genes are up-regulated (Fig. 1D). We did not observe a statistically significant difference in the expression of *BTD*. This is expected, because biotinidase is a constitutively expressed enzyme [9]. We observed little up- or down-regulation of the biotin-dependent carboxylases, the enzymes involved in the pathways of carboxylase metabolites or in the urea cycle enzymes under biotin deficient conditions, with the exception of *N*-acetylglutamate synthetase (Fig. 2D).

We did observe many up-regulated enzymes in multiple neurological pathways involving neuronal excitation and activity and neurological morphology (Table 1D). These changes may be important for the pathogenic changes observed in the symptomatic individual. In addition, we also observe changes in the gene expression observed in the KO Day8/Day1 comparison that may be indicative of the cellular immunity alterations seen in human biotinidase deficiency [10].

Lymphocytic infiltrations have been reported in the brain of a child that died prior to making the diagnosis of biotinidase deficiency, but these changes were obtained at autopsy [11]. Given our results, the significance of lymphocytic infiltration is interesting, but these changes in this child may be more consistent with a terminal event. We did not see any indication of lymphocytic infiltrations in the brains of symptomatic KO mice that we reported previously [7]. The gene up-regulation we observed may have been an early stage in the process of mobilizing lymphocytes in symptomatic animals.

The absolute magnitude of the changes in gene transcription may not indicate true differences on their effect on metabolic pathways. To determine the effect(s) that these changes in transcription have on a pathway will require a comprehensive understanding of biochemistry and physiology of the involved pathways. Although we are currently in a position to examine the degree of up- or down-regulation of specific genes or common pathways, interpretation of their effects will require more powerful integrative computer programs to analyze these complex metabolic pathways and their interactions.

Biotinidase deficiency is a monogenic deficiency that in the untreated individual can produce clinical features involving primarily the neurological and cutaneous systems. We have only examined the neurological system in this study, but clearly, we see that multiple genes and pathways are affected. We may have been naïve in believing that we would uncover or discover a specific gene or group of genes that would help answer some important questions, such as why there is such variability in expression of the disorder in the same family or why some untreated individuals develop symptoms during adolescence, adulthood or even remain asymptomatic.

We hoped that these preliminary studies of gene expression in the various scenarios would provide us with clues or directions for areas of further study. However, the changes in expression of various genes or gene pathways have not necessarily provided us with specific answers. Moreover, the gene expression alterations may not indicate direct effects, but possibly more down-stream effects, thereby making interpretation even more difficult.

These studies together with appropriate computerized programs may also provide us with a method for understanding the biochemistry and pathophysiology in various organ systems of other monogenetic inherited metabolic disorders [12]. The current study is limited by the lack of adequate programs to analyze these data. However, our data is accessible for future analysis. As the interest in using these technologies increases and our ability to understand the interrelationships of various biological and physiological pathways and mechanism improves, we will surely be able to interpret better these data and understand the various affected and interactive pathways.

Our intent in performing these studies was to determine if we could find any genes or pathways that could provide us with further insights into changes that are occurring when KO animals become symptomatic. As we have stated, this is a preliminary study. However, we believe that this study is important in informing other investigators of inherited metabolic diseases about some of the strengths and weaknesses of this approach for their research.

This study represents an attempt to narrow the search for mechanistic explanations of biotinidase deficiency. We are certainly not the first to use such an approach with the understanding that the results may not provide specific answers without further advancements in bioinformatics. These preliminary analyses have provided us with some new information that confirms existing knowledge, and it may serve as a guide for future studies targeted at pathways implicated here. The development of new algorithms and programs examining these mechanisms and pathways of the central nervous system and immune system may better explain the role of biotinidase and the pathogenesis of biotinidase deficiency.

## Figures and Tables

**Table 1:** Up-regulated and down-regulated gene expression pathways.

Diseases or functions annotation	*p*-Value	# of Genes	Genes (↑ Upregulated, or ↓ Downregulated)
A. KO/WT Day1			
Targeting of mossy fibers	6.51E-06	2	EN1 ↑, EN2 ↑
Quantity of mesencephalic neurons	3.89E-05	2	EN1 ↑, EN2 ↑
Size of inferior colliculus	3.89E-05	2	EN1 ↑, EN2 ↑
Lack of cerebellum	1.36E-04	2	EN1 ↑, EN2 ↑
Growth of pituitary gland	2.89E-04	2	CGA ↑, PITX1 ↑
Abnormal morphology of somatic nervous system	4.23E-04	2	HOXB5 ↓, HOXB6 ↓
Survival of dopaminergic neurons	4.23E-04	2	EN1 ↑, EN2 ↑
Development of colliculus	2.60E-03	1	EN1 ↑
Development of nucleus accumbens	2.60E-03	1	ALDH1A3 ↓
Hypertrophy of thyrotropes	2.60E-03	1	CGA ↑

B. WT Day8/Day1			
Development of head	3.43E-04	15	BARHL2 ↓, CFH ↑, DCLK1 ↑, DKK1 ↓, FEZF1 ↓, IRX6 ↓, LHX1 ↓, LRP2 ↓, NKX2-1 ↓, PITX1 ↓, SLC17A7 ↑, SOX4 ↓, SRC ↑, THRB ↑
Formation of brain	2.05E-03	13	BARHL2 ↓, DCLK1 ↑, DKK1 ↓, FEZF1 ↓, IRX6 ↓, LHX1 ↓, LRP2 ↓, NKX2-1 ↓, SLC17A7 ↑, SOX4 ↓, SRC ↑, THRB ↑
Formation of forebrain	2.55E-03	8	DCLK1 ↑, DKK1 ↓, FEZF1 ↓, IRX6 ↓, LRP2 ↓, NKX2–1 ↓, SRC ↑
Development of interneurons	3.83E-03	2	CADPS2 ↓, NKX2–1 ↓
Growth of pituitary gland	4.75E-03	2	PITX1 ↓, THRB ↑
Maturation of neurons	8.06E-03	3	DISC1 ↓, GPR37L1 ↑, KLF9 ↑
Formation of cerebellum	8.94E-03	5	LHX1 ↓, SLC17A7 ↑, SOX4 ↓, SRC ↑, THRB ↑
Formation of cells	9.40E-03	4	BARHL2 ↓, NCAM2 ↑, NKX2–1 ↓, SLC17A7 ↑
Abnormal morphology of cerebellum fissure	1.06E-02	1	CADPS2 ↓
Abnormal morphology of infundibular stem	1.06E-02	1	NKX2-1 ↓

C. KO/WT Day 8			
Abnormal morphology of mammillary body	2.58E-05	2	FOXB1 ↑, OTX2 ↑
Abnormal morphology of hypothalamus	4.11E-05	3	FOXB1 ↑, OTX2 ↑, SIM1 ↑
Abnormality of nervous system	6.74E-05	3	DRD1 ↓, NHLH2 ↑, TPH2 ↑
Formation of neurons	3.36E-04	3	EBF3 ↑, OTX2 ↑, PHOX2B ↑
Formation of forebrain	4.78E-04	5	DRD1 ↓, EBF3 ↑, OTX2 ↑, RARB ↓, SIM1 ↑
Formation of brain	5.06E-04	7	DRD1 ↓, EBF3 ↑, FOXB1 ↑, OTX2 ↑, PHOX2B ↑, RARB ↓, SIM1 ↑
Abnormal morphology of forebrain	5.62E-04	5	DRD1 ↓, FOXB1 ↑, OTX2 ↑, RXRG ↓, SIM1 ↑
Morphology of nervous system	6.73E-04	9	CGA ↑, DRD1 ↓, FOXB1 ↑, MAL ↑, OTX2 ↑, RARB ↓, RXRG ↓, SIM1 ↑, TPH2 ↑
Quantity of cells	6.89E-04	7	CGA ↑, DRD1 ↓, NHLH2 ↑, OTX2 ↑, PENK ↓, SIM1 ↑, TPH2 ↑
Quantity of central nervous system cells	7.57E-04	4	CGA ↑, NHLH2 ↑, PENK ↓, TPH2 ↑

D. KO Day8/Day1			
Action potential of motor endplates	1.35E-03	4	COL13A1 ↑, DNAJC5 ↑, PRKCQ ↑, RIMS1 ↑
Cell movement of mononuclear leukocytes	1.50E-03	7	CD44 ↑, IL11RA ↑,IL12B ↑, ITGA4 ↑, PRKCQ ↑, SELP ↑,SELPLG ↑
Excitation of brain cells	1.80E-03	6	CHRM1 ↑, CHRNA7 ↑, HCRT ↑, KCNIP2 ↑, KCNQ2 ↑, TRPV4 ↑
Excitation of neurons	1.95E-03	8	CHRM1 ↑, CHRNA7 ↑, HCRT ↑, KCNIP2 ↑, KCNQ2 ↑, KISS1 ↑, PRKCG ↑, TRPV4 ↑
Quantity of neuroendocrine cells	2.47E-03	8	AVPR1B ↑, CGA ↑, ESR1 ↑, LIF ↑, NOTCH2 ↑, NTN1 ↑, TFAP2A ↑, THRB ↑
Lymphocyte migration	2.52E-03	6	CD44 ↑, IL12B ↑, ITGA4 ↑, PRKCQ ↑, SELP ↑, SELPLG ↑
Cell movement of T lymphocytes	2.87E-03	5	CD44 ↑, ITGA4 ↑, PRKCQ ↑, SELP ↑, SELPLG ↑
Infiltration of cells	3.09E-03	8	AGER ↑, CD44 ↑, CRYAB ↑, CSF3 ↑, IL11RA ↑, ITGA4 ↑, MBP ↑, PRKCQ ↑
Abnormal morphology of cranial nerve ganglion	4.69E-03	8	COL2A1 ↓, ERBB3 ↑, ERBB4 ↑, NTF4 ↑, POU4F1 ↓, SIX1 ↑, TFAP2A ↑, VTI1A ↑
Abnormal morphology of trigeminal ganglion	5.42E-03	7	ERBB3 ↑, ERBB4 ↑, NTF4 ↑, POU4F1 ↓, SIX1 ↑, TFAP2A ↑, VTI1A ↑
